# Cell surgery and growth factors in dry age-related macular degeneration: visual prognosis and morphological study

**DOI:** 10.18632/oncotarget.10442

**Published:** 2016-07-06

**Authors:** Paolo Giuseppe Limoli, Celeste Limoli, Enzo Maria Vingolo, Sergio Zaccaria Scalinci, Marcella Nebbioso

**Affiliations:** ^1^ Low Vision Research Centre of Milan, Milan, Italy; ^2^ Department of Ophthalmology, A. Fiorini Hospital, Terracina, Polo Pontino, Sapienza University of Rome, Rome, Italy; ^3^ Departement of Ophthalmology, Glaucoma and Low Vision Study Center, University of Bologna, S. Orsola–Malpighi Hospital, Bologna, Italy; ^4^ Department of Sense Organs, Faculty of Medicine and Odontology, Sapienza University of Rome, Rome, Italy

**Keywords:** age-related macular degeneration (AMD), cell autologous graft, growth factors (GFs), limoli retinal restoration technique (LRRT), suprachoroidal graft, Gerotarget

## Abstract

**Background:**

The aim of this research was to study the overall restoration effect on residual retinal cells through surgically grafted autologous cells onto the surrounding tissue, choroid and retina in order to produce a constant secretion of growth factors (GFs) in dry age-related macular degeneration (AMD) patients.

**Results:**

6 months after surgery, several values were statistically significant in the group with higher RTA. Also patient compliance analysis (PCA) in relation to functional change perception appeared to be very good.

**Methods:**

Thirty-six eyes of 25 patients (range 64-84 years of age) affected by dry AMD were included in study, and divided in two groups by spectral domain-optical coherence tomography (SD-OCT): group A with retinal thickness average (RTA) less than 250 microns (μm) and group B with RTA equal to or more than 250 μm. Adipocytes, adipose-derived stem cells from the stromal-vascular fraction, and platelets from platelet-rich plasma were implanted in the suprachoroidal space. Particularly, the following parameters were evaluated: best corrected visual acuity (BCVA) for far and near distance, retinal thickness maps, scotopic and photopic electroretinogram (ERG), and microperimetry (MY). All statistical analyses were performed with STATA 14.0 (Collage Station, Texas, USA).

**Conclusions:**

The available set of GFs allowed biological retinal neuroenhancement. After 6 months it improved visual performance (VP), but the increase was better if RTA recorded by OCT was higher, probably in relation to the presence of areas with greater cellularity.

## INTRODUCTION

Age-related macular degeneration (AMD) is a heterogeneous clinical condition in which retinal degeneration occurs predominantly in the macula and leads to impairment of central visual acuity [1, 2]. AMD, the leading cause of blindness in people aged 55 years or older in developed countries, occurs in two general forms: 1) Wet AMD involving choroidal neovascularization with subsequent bleeding and fluid exudation; 2) Dry AMD, which involves a constellation of clinical features that can include: drusen, pigment clumping, abnormalities of the retinal pigment epithelium (RPE), and geographic atrophy (GA) [1–3]. The latter can begin as a thinning of the RPE and lead subsequently to an atrophic change in the macula with loss of overlying photoreceptors [2–4]. As a result, there is a reduction of best corrected visual acuity (BCVA), retinal sensitivity, retinal bioelectric activity, and then damage to visual performance (VP). Nowadays, spectral domain-optical coherence tomography (SD-OCT) has been added to electroretinographic exams on retina for more accurate morphological and functional studies [5].

Moreover, dry AMD accounts for 80% of all intermediate and advanced forms of the retinal disease and no effective treatment for progressive vision loss is available to date. On the other hand, the retina is particularly susceptible to oxidative damage due to its high oxygen demand and constant exposure to light. It is known that smoking may slow down choroidal blood flow, promoting ischemia, hypoxia, and microinfarctions, resulting in a reduction in macular pigments and in RPE damage. Oral supplement of antioxidants and smoking cessation can help to prevent or counter AMD development and progression [1–3]. However, dry AMD progression reduces VP causing discomfort to patients who become increasingly more visually impaired [3–4].

Consequently, the idea of using systemic or local injection of stem/progenitor or reparatory cells in the area of injury to treat multiple chronic disorders has received close attention in the last decade [6]. Unfortunately, the appropriate surgical procedure to graft cells in the subretinal space appears to be very complex and expensive, as well as potentially dangerous for the eye, as it undergoes invasive surgery. Instead, embryonic stem cells are not always ethically acceptable and not entirely free from the risks related to the use of immunosuppressive therapy [7].

Nevertheless, there is another aspect linked to the properties of growth factors (GFs) which have been known for many years and specifically to their ability to curb the evolution of retinal dystrophies in experimental models, as demonstrated by several studies. GFs and neurotrophins such as basic fibroblast growth factor (bFGF), neural growth factor (NGF), ciliar neurotrophic factor (CNTF) and brain-derived neurotrophic factor (BDNF), can significantly slow down retinal degeneration and cell death in animal models [7–10].

Based on these concepts, the efficacy of autologous fat transplantation was demonstrated by Filatov [11] work and Pelaez [12] first, and by Meduri et al. [13] and Limoli et al. later [14].

Our cellular autograft, defined Limoli Retinal Restoration Technique (LRRT), uses three different cell types that are able to produce adequate GFs in terms of both quantity and quality:

Adipose stromal cells derived from orbital fat [15].

Platelets (PLT) derived from platelet-rich plasma (PRP) [16, 17].

Adipose-derived stem cells (ADSCs) included in the stromal-vascular fraction (SVF) of adipose tissue [18].

Cellular autograft efficacy in dry AMD was demonstrated by Limoli et al. in preliminary works [4, 19–21]. Indeed, to curb the worsening of retinal dry AMD, we considered employing two important elements:

neurotrophic GFs to slow down RPE cells and photoreceptor apoptosis;

angiotrophic GFs for therapeutic purposes to improve choroidal flow; in fact, the impairment of the latter is belived to be one of the pathogenetic factors of AMD.

Consequently, the rationale of our study was focused on cell therapy, through autologous cells surgically grafted in the suprachoroidal space, in order to achieve a costant production of GFs at the chorioretinal level. Besides, to evaluate the prognosis of dry AMD patients treated with LRRT, we assumed that a greater number of residual cells leads to greater interaction between GFs and chorioretinal cellular membrane receptors, more intense cellular activity and, ultimately, improvement of VP. In other words, we hypothesized that if retina is thinner and more atrophic, it is harder to obtain functional neuroenhancement through cell therapy. In order to focus on, we chose SD-OCT for the preliminary analysis of retinal thickness average (RTA) in cell therapy with LRRT on patients affected by dry AMD.

## RESULTS

In the study we included 36 eyes (21 right and 15 left eyes) of 25 patients with dry AMD (9 men and 16 women), aged between 64 and 84 years of age (75.96 years on average) (SD ±6.13) (Table 1). Prior to cell autograft with LRRT, values of RTA and all components of ERG were recorded at time 0 (T0).

**Table 1:** Baseline demographic and clinical characteristics by retinal thickness average (RTA) in patient group A (RTA<250 microns) and group B (RTA ≥250 microns)

Patient data	Group A	Group B	Total
Number of patients (*N*)	*N* = 10	*N* = 15	*N* = 25
Female (%)	8 (80.0)	8 (53.3)	16 (64.0)
Male (%)	2 (20.0)	7 (46.7)	9 (36.0)
Number of eyes (right/left)	14 (8/6)	22 (13/9)	36 (21/15)
Average age (±SD)	77.6 (6.6)	74.87 (5.8)	76.0 (6.13)

Based on RTA, all the eyes were divided into two groups according to normative data for macular thickness by high-definition SD-OCT (Table 1): [5]

**Figure 1:**
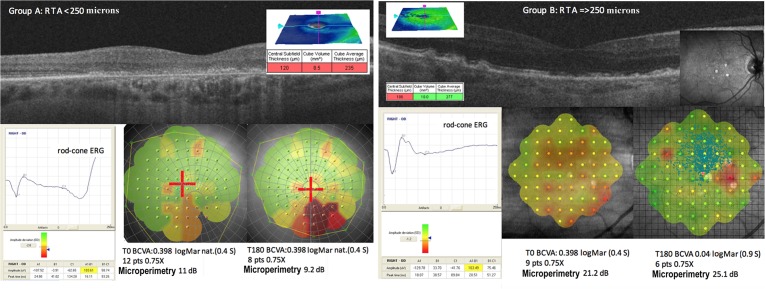
The level of scotopic (rod-cone or maximal) electroretinogram (ERG) is different in A and B groups depending on retinal thickness Case report of group A (left panel). The retinal thickness average (RTA) measured by optical coherence tomography (OCT) is very low (235 microns). At 180 days post surgery we obtained a slight improvement in close-up visus. Case report of group B (right panel). The RTA is good (277 microns) and the various cell layers are well represented in the OCT. At 180 days post surgery we obtained a good improvement of best corrected visual acuity (BCVA) and close-up visus (in logMAR and Pts respectively) without magnification change. Also the sensitivity recorded with microperimetry (MY) increased in the areas with good cellularity.

- Group A, where RTA was less than 250 microns (μm), with 14 eyes, 10 patients (2 men and 8 women), mean age of 77.6 years (range 64-84 years of age, SD ±6.6) (Figure 1, left panel).
- Group B, where RTA was equal to or greater than 250 μm, with 22 eyes, 15 patients (7 men and 8 women), mean age of 74.87 years (range 64-84 years of age, SD ±5.8) (Figure 1, right panel).

No significant differences were found between the two groups either in terms of age (*p* = 0.284) or gender (p = 0.174) distribution.

### Correlation with retinal thickness average (RTA) and electroretinogram (ERG)

**Figure 2:**
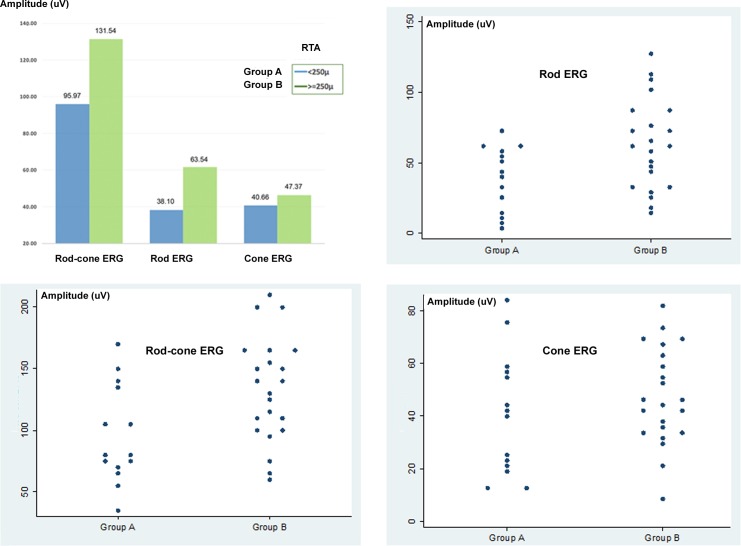
Electroretinogram (ERG) and retinal thickness average (RTA) in dry age-related macular degeneration (AMD) at time 0 (T0) Correlation between RTA and electrical activity (in μVolts) appears to be highly significant in relation to scotopic ERG: rod-cone or maximal ERG in group A (*p* = 0.025) and rod ERG in groups A (*p* = 0.006) and B (*p* = 0.011). Corresponding scatter plot graph.

ERG-recorded electrical activity (T0) in group A (Rod ERG = 38.1, Rod-cone ERG = 95.97, Cone ERG = 40.66) was lower than in group B (Rod ERG = 63.54, Rod-cone ERG = 131.54, Cone ERG = 47.37) (Figure 2). RTA was positive correlated with Rod-cone ERG in group A (r = 0.59, *p* = 0.025), but not in group B (r = −0.29, *p* = 0.196). RTA was positive correlated with Rod ERG in group A (r = 0.69, *p* = 0.006) and group B (r = 0.53, *p* = 0.011). (Figure 2).

### Visual functional characteristics and patient compliance analysis (PCA)

The visual functional characteristics in patients with dry AMD and the average values recorded before (T0) and 6 months (T180) after cell autograft by LRRT are shown in Table 2. BCVA was measured by early treatment diabetic retinopathy study (ETDRS) charts at 4 meters in logarithm of the minimum angle of resolution (logMAR) units and visual acuity for near vision (close-up) in points (Pts) indicating the necessary zoom factor (X).

**Table 2:** Mean values recorded before (T0) and 6 months (T180) after surgery with Limoli Retinal Restoration Technique (LRRT) for retinal thickness average (RTA) of group A (RTA<250 microns) and group B (RTA ≥250 microns) and statistical significance

Parameter	Group A		*P* value*	Group B		*P* value*
	T0	T180		T0	T180	
	Mean (SD)			Mean (SD)		
BCVA (logMAR)	0.62 (0.57)	0.58 (0.56)	0.465	0.59 (0.39)	0.41 (0.3)	0.001
Close-up visus (Pts)	12.14 (12.3)	8.86 (5.36)	0.083	8.68 (2.87)	8.27 (3.33)	0.33
Close-up visus X	5.13 (4.44)	5.13 (4.08)	1.000	3.11 (3.39)	2.92 (3.48)	0.03
Retinal sensitivity	7.65 (4.83)	8.41 (4.75)	0.237	16.82 (3.2)	18.4 (3.4)	0.01

Parameter	Group A		Group B		*P* value*
	Mean changes (SD)	%	Mean changes (SD)	%	
BCVA (logMAR)	0.033 (0.17)	5.16%	0.18 (0.23)	30.51%	0.04
Close-up visus (Pts)	3.29 (7.35)	27.10%	0.41 (2.02)	4.72%	0.09
Close-up visus X	0 (1.95)	0%	0.19 (0.49)	6.10%	0.43
Retinal sensitivity	0.76 (2.51)	9.93%	1.59 (3.34)	9.45%	0.50

* Calculated by Mixed regression model taking into account multiple eyes for the same subjects. BCVA= best corrected visual acuity for far distance measured by early treatment diabetic retinopathy study (ETDRS) charts at 4 meters in logarithm of the minimum angle of resolution (logMAR) units; close-up visus = visual acuity for near vision in points (Pts) measured by magnifying system and necessary magnification, X = necessary magnification; Retinal sensitivity = measured by microperimetry (MY) in decibel (dB); SD = standard deviation.

**Figure 3:**
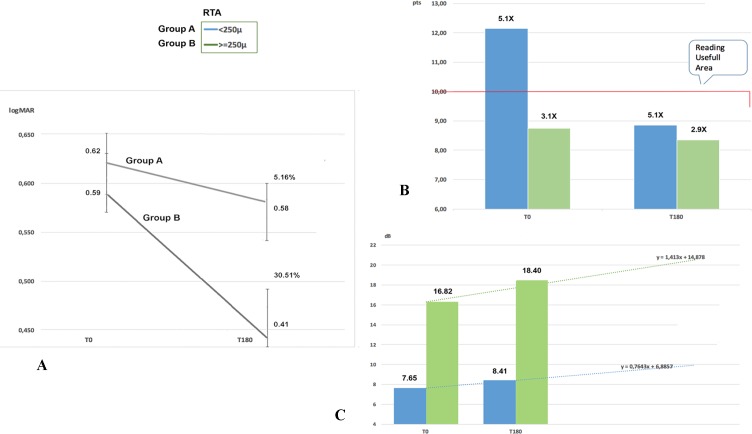
Values recorded from time 0 (T0) to time at 6 months (T180) after Limoli Retinal Restoration Technique (LRRT) Panel **A.** Best corrected visual acuity (BCVA) recorded a slight increase (5.16%) in group A and a greater increase (30.51%) in group B. Panel **B.** After LRRT, close-up visus (in Pts) evaluated by magnification reaches or maintains values below 10 points (Pts) in both groups. Panel **C.** The average retinal sensitivity (in dB) recorded by microperimetry (MY) 6 months after cellular autograft increases in both groups: +6.88% in group A and +15.23% in group B. Retinal thickness average (RTA).

**Figure 4:**
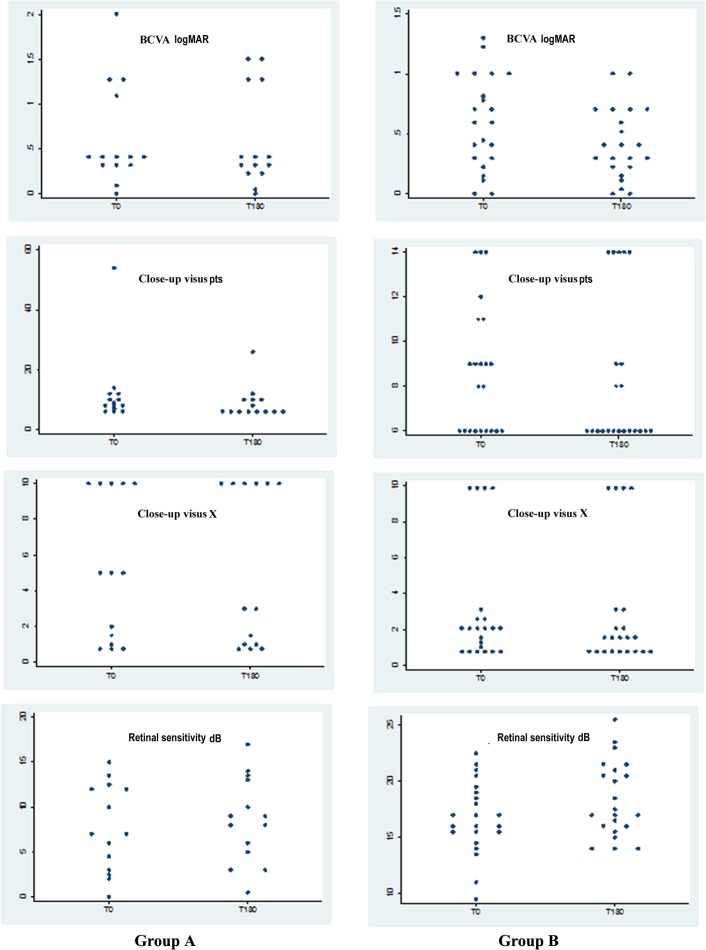
Corresponding scatter plot graphs of the values recorded from time 0 (T0) to time at 6 months (T180) after Limoli Retinal Restoration Technique (LRRT) Best corrected visual acuity (BCVA) (in logMAR); close-up visus (in Pts) evaluated by magnification reaches or maintains values below 10 Pts in both groups; retinal sensitivity (in dB).

BCVA increased in group A from 0.621 logMAR to 0.588 logMAR and in group B from 0.59 logMAR to 0.41 logMAR. Mean variation was significantly lower in A (0.033 logMAR SD 0.17) than in B (0.18 logMAR SD 0.23) (Figure 1, 3A, 4) (Table 2).

Close-up visus evaluated with magnifying system increased in both groups from T0 to T180. In group A, it went from 12.14 to 8.86 Pts with the same magnification (X 5.1) and a mean increase of 3.29 Pts (SD 7.35). In group B, visual acuity went from 8.68 to 8.27 Pts with slightly lower magnification (from 3.1 to 2.9 X), equivalent to an increase of 0.41 Pts (SD 2.0) (Figure 1, 3B, and 4) (Table 2).

Average retinal sensitivity with microperimetry (MY) increased in both groups. The mean improvement in retinal sensitivity was 0.76 dB (SD 2.5), increasing from 7.65 dB to 8.41 dB in group A, and 1.59 dB (SD 3.34), increasing from 16.82 dB to 18.4 in group B (Figure 1, 3C, and 4) (Table 2).

Patient compliance analysis (PCA) showed that, at 6 months post surgery, 19 out of 36 eyes (52.78%) recorded better vision, 14 out of 36 (38.89%) no change in the functional situation, and 3 out of 36 (8.33%) a worsening (Table 3). Among the 19 eyes of the patients who noted an improvement at 6 months post surgery, 5 (26.3%) belonged to group A and 14 (73.7%) to group B.

No adverse effects were reported in any case and mean values of the intraocular pressure recorded before and after surgery did not change significantly.

**Table 3:** Patient compliance analysis (PCA) shows that, at 6 months post surgery, 19 of 36 eyes (52.78%) recorded better vision, 14 (38.89%) no change in functional situation, and 3 (8.33%) a worsening

PCA	Group A	Group B	Total
Number of eyes	14	22	36
Improved (%)	5 (35.71)	14 (63.64)	19 (52.78)
Unchanged (%)	7 (50)	7 (31.82)	14 (38.89)
Worse (%)	2 (14.29)	1 (4.55)	3 (8.33)

Among the 19 eyes of patients who noted an improvement at 6 months post surgery, 5 (26.3%) belong to group A and 14 (73.7%) to group B.

## DISCUSSION

We considered employing GFs for therapeutic purposes in order to improve choroidal flow, slow down RPE cells and photoreceptor apoptosis, and ultimately curb retinal worsening in dry AMD patients treated with LRRT. Moreover, we assumed that a greater number of residual cells could lead to a greater interaction between GFs and chorioretinal cellular membrane receptors, to more intense cellular activity and, ultimately, to an improvement of VP. Since GFs and neurotrophins usually have a short half-life, the promotion of retinal cell survival needs continuous and frequent administration of these factors. Their short half-life and the consequent need to perform frequent intravitreal injections with the ensuing complications have not allowed therapeutic use in humans so far [8–10].

each eye of our subjects received cellular autograft according to LRRT, after the harvest of SVF with ADSCs and Platelet Rich Plasma, an innovation of Pelaez-Meduri's intervention [4, 12, 13, 19]. They used a transplantation of autologous fat in the subscleral space, obtaining relevant but transient results [5, 19]. The goals of LRRT, that is the grafting of the suprachoroidal adipose pedicle, of ADSCs in SVF and intrapeduncolar PRP were to:

1. Promote vascular pedicle fat engraftment with the underlying choroid;
2. Enhance pedicle fat original vascularization in order to ensure its volume and survival;
3. Initiate a regenerative start-up of all the retinal elements through the secretion of paracrine GFs.

Knowing the amount of cells in the retina could be useful for both rehabilitators and surgeons in order to plan a cell therapy with an acceptable predictive power in low vision patient with dry AMD. Moreover, we observed that cone-rod ERG and rod ERG present a highly significant correlation with RTA, while cone ERG does not, since the main functional expression of fovea appears to be compromised also with still regular macular volumes in dry AMD, at least in the initial stages (Figure 2) [4, 5, 19]. Residual retinal trophism in dry AMD, value with RTA, may represent a prognostic criterion for the indication of cellular autograft with LRRT, since better outcomes are found increasingly in group B. We already saw the positive action on retinal electrical activity in our previous study, and the current study has shown that scotopic ERG, BCVA and sensitivity detected by MY appear to be more intense in thicker retinas (Table 2, Figure 2, 3, and 4) [4, 19]. On the contrary, natural evolution shows inevitable visual impairment, according to clinical events [1–3].

LRRT allows the grafting of 3 autologous components and each of the grafted elements has its own specific assets in GFs useful from a regenerative point of view.

*Fat cells* of the pedicle grafted and contained in the suprachoroidal space produce: basic fibroblast growth factor (bFGF), epidermal growth factor (EGF), insulin-like growth factor-1 (IGF-1), interleukin (IL), transforming growth factor-β (TGF-β), pigment-epithelium-derived factor (PEDF), and adiponectin [22].

*ADSCs* produce: bFGF, vascular endothelial growth factor (VEGF), macrophage colony-stimulating factor (M-CSF), granulocyte-macrophage colony-stimulating factor (GM-CSF), placental growth factor (PlGF), TGF-β, hepatocyte growth factor (HGF), IGF-1, IL, and angiogenin [15].

*Platelets* produce: platelet-derived growth factor (PDGF), IGF-1, TGF-β, VEGF, bFGF, EGF, platelet-derived angiogenesis factor (PDAF), and thrombospondin (TSP) [23–25].

Therefore, following the complex process of GF-receptor formation, a limited number of second messengers are generated inside the target cell. These, in turn, control a series of biochemical pathways in the cell by regulating enzyme activity and transcription factors, through a cascade of phosphorylation events [19–25]. Upon reaching the cell nucleus, the signals determine changes in the response of genes, increasing the production of proteins, enzymes, and cytokine transcription which all play a role in cell trophic control, as demonstrated by the increase in scotopic ERG-recorded electrical activity [19–25].

The main function of GFs is the external control of the cell cycle from the quiescent cell phase, G0 phase, to the entry of the cell in the G1 phase of growth. In addition, they can regulate the triggering of mitosis, cell survival, migration, and cell differentiation. Some factors, such as VEGF, bFGF, angiogenin, PDAF, PlGF, PDGF, EGF, and TGF-β promote endothelial regeneration and may contribute to choriocapillaris reperfusion [16, 17, 25, 26]. VEGF, introduced with PRP, stimulates ADSC proliferation which in turn promotes both autologous fat and adipocytes survival [21, 26, 27]. PEDF, similarly to bFGF, has a neurotrophic action on photoreceptors. Some factors, such as EGF, act on Müller cells inducing endogenous bFGF transcription and strengthen the protective action of these cells on the retina nerve cells; EGF also stimulates ADSCs to increase their secretory activity [30, 29].

TGF-β^32^, Moreover, M-CSF, GM-CSF, and IL have both an anti-inflammatory and chemotactic action on macrophages which contribute to the removal of intraretinal cell debris and to the function normally carried out by RPE [34, 35].

From the results of our study, LRRT may result, directly and indirectly, in increased choroidal perfusion and greater trophism of the photoreceptors through GF-receptor interactions and stimulation mediated by Müller cells and RPE cells. Subsequently, these events are believed to allow more intense cellular activity as demonstrated by the improvement in electrical activity [4, 19]. It is logically supposed that the bond between cells and GF could be the necessary link in the chain of events responsible for an improvement of VP in dry AMD patients. Obviously, poor tissue cellularity would not allow the desired therapeutic effect due to scarce GF-membrane receptor interactions.

Ultimately, our results allow us to conclude that the technique offers some advantages in providing benefits to patients suffering from dry AMD. Vice versa, the surgical procedures available to date are more complex and only temporarily effective [6–13]. Obviously, our studies also have limits to overcome, such as further validation with a greater number of patients and histopathological findings that could provide scientific evidence for the results. Also a control group will be included in the next research study to exclude a placebo effect.

Therefore, we will continue our studies to provide useful updates to the scientific world on this disease which is responsible for growing disability in the modern world due to aging and will therefore have a growing burden on society.

## CONCLUSIONS

Cell therapy by autologous cell graft in the suprachoroidal space according to LRRT seems to afford a functional improvement in retinal residual cells, proportional to their condition, with positive consequences on patient VP. The preliminary reading of OCT may allow rehabilitators and surgeons to plan this type of surgery/therapy in patients with dry AMD. Including in severe cases the contribution of GFs to the residual cells of the retina allows the optimization of rehabilitation and recovery of lost capacity with appropriate magnifying systems.

## PATIENTS AND METHODS

The tenets of the Declaration of Helsinki were observed, and written informed consent approval by the Ethics Committee of the Low Vision Academy was obtained.

For this purpose, we included 36 eyes of 25 patients (16 women and 9 men), with an average age of 75.96 years (range from 64 to 84 years of age) affected with dry AMD.

They were identified and enrolled according to several characteristics. The inclusion criteria were:

- Caucasian patients classified as well-nourished;  
- age ranging from 64 to 84 years;
- dry AMD diagnosis with SD-OCT, fundus autofluorescence imaging, and angiography in the presence of drusen and irregularities of RPE in at least 1 eye;  
- well-preserved extrafoveal areas;  
- measurable visual acuity not influenced by lens opacity;  
- normal intraocular pressure;  
- acceptance of the clinical protocol by signing the informed consent.

The criteria for exclusion from the study due to possible cross interference with the test were as follows:

- patients with signs of exudative AMD;
- myopia or hypermetropy with spherical equivalent of ≥ 6 diopters;
- cataract, chorioretinal and neovascular membrane-associated disorders, macular pucker, uveitis, etc.;
- other ocular disorders, such as glaucoma, optic neuritis, ocular trauma, high refractive errors, etc.;
- insufficient compliance in individuals affected by medical problems, such as hypovitaminoses, multiple sclerosis, epilepsy, Parkinson's disease, diabetes mellitus, hypertension, vasculitis, renal and hepatic diseases, gastrointestinal malabsorption, hypothyroidism, malignant neoplasias, and other systemic acute or chronic diseases.

The eyes were retrospectively divided into two groups according to RTA: group A in which RTA was less than 250 μm, and group B in which RTA was equal to or greater than 250 μm (Figure 1). For each patient we evaluated the visual acuity for far and near distance at T0. BCVA was measured with ETDRS charts at 4 meters in logMAR and visual acuity for near vision, close-up, in Pts indicating the necessary zoom factor (X). The diagnosis of dry AMD was confirmed by Nidek F10 confocal scanning laser ophthalmoscope (Nidek Inc, Fremont, CA), Cirrus 5000 SD-OCT (Carl Zeiss Meditec AG, Jena, Germany), and Maia 100809 MY (CenterVue S.p.A., Padua, Italy). Therefore, we recorded electrical cell activity through scotopic and photopic ERG (ocular electrophysiology electromedical system, Retimax; C.S.O. S.r.l., Scandicci, Italy) according to the standards set in 2009 by the International Society for Clinical Electrophysiology of Vision (ISCEV) [19].

Fat tissue was collected and purified from the abdominal subcutaneous layer of patients according to the Lawrence and Coleman [20] technique. Briefly, 10 mL of fat tissue was harvested manually from each patient with a 3 mm blunt cannula (Mentor, Santa Barbara, CA) connected to a Luer-LokTM syringe (BD Biosciences, Franklin Lakes, NJ). Pure SVF of fat tissue was separated from blood, fat, oil, and liquid after 3′ of centrifugation at 3200 rpm at 1200 G. For PRP preparation, 8 mL of human peripheral blood was collected in a Regen-BCT tube (RegenKit; RegenLab, Le Mont-sur-Lausanne, CH). The collected blood was centrifuged for 5′ at 1500 G. In the LRRT variant, the following changes were made to increase autologous fat graft survival, to trigger ADSC proliferation in order to promote increased choroidal perfusion, and to obtain a more complete modulation of the action of factors secreted only by fat [15, 17, 19–21].

1. The distance between the grafted autologous cells and choroid was reduced by deep sclerectomy to favor the paracrine secretion of the autologous cells into the choroidal flow.
2. For the same reason, the area of contact between the stalk and choroid was expanded.
3. The suprachoroidal pocket was built to accommodate the graft and saturated with a mixture of ADSCs and SVF obtained according to the Lawrence and Coleman technique [20].
4. The adipose pedicle was infiltrated with PRP gel obtained by centrifugation of the blood material, separation of the component, and its platelet degranulation [16].

The details are as follows:

- A deep scleral door of about 5 mm was opened by radial hinge in the infero-temporal quadrant at 8 mm from the limbus. The sclerectomy must be deep enough to allow viewing of the slate color of the choroid.
- Orbital fat was extracted from a gap above the inferior oblique muscle. The extracted pedicle fat must be sufficient to ensure the survival of the vascular scaffold after its location.
- The flap of autologous fat was laid gently on the bed and sutured with choroidal vicryl 6/0 at the proximal edge of the door.  
- The scleral flap was then secured to avoid compression on the fat pedicle or on its nutrient vessels.  
- The remaining space between the autologous fat graft, choroid, and scleral flaps was saturated with 0.5 cc of SVF, previously prepared with ADSCs by venflon inserted into the scleral pocket.  
- Then, the stroma of the peduncle was infiltrated with 1 cc of PRP using a 25-gauge cannula.

Therefore, an autograft consisting of fat cells, ADSCs from SVF, and PRP was obtained.

The functional analysis was then repeated after 180 days. Particularly, we considered BCVA and close-up visus after visual rehabilitation and the necessary magnification factor, retinal sensitivity, PCA, RTA, and ERG activity in both groups.

### Statistical Analysis

Student's *t-test* was used to compare mean age between the two study groups. Gender distribution was compared by *chi square* test. In order to take into account multiple eyes for the same patients, a Mixed linear regression model was used to evaluate the difference in this parameter at T0 between the two groups and the variation at T0 and T180, and to compare the mean change between the two groups. A *p* value of < 0.05 was considered statistically significant. All statistical analyses were performed with STATA 14.0 (Collage Station, Texas, USA).

## Abbreviations

ADSCs: adipose-derived stem cells
AFT: autologous fat transplantation
AMD: age-related macular degeneration
BCVA: best corrected visual acuity for far distance
bFGF: basic fibroblast growth factor
BDNF: brain-derived neurotrophic factor
BM: Bruch membrane
CC: choriocapillaris
close up visus: visual acuity for near vision
CNTF: ciliar neurotrophic factor
dB: decibel
EGF: epidermal growth factor
ERG: electroretinogram
ETDRS: early treatment diabetic retinopathy study
G0: quiescent cell phase
G1 phase: cell entry in the growth phase
GF: growth factor
GM-CSF: granulocyte-macrophage colony-stimulating factor
HGF: hepatocyte growth factor
IGF-1: insulin-like growth factor-1
IL: interleukin
logMAR: logarithm of the minimum angle of resolution
LRRT: Limoli Retinal Restoration Technique
M-CSF: macrophage colony-stimulating factor
MY: microperimetry
μm: microns
mV: micronVolts
NGF: neural growth factor
NS: not statistically significant
PCA: patient compliance analysis
PDAF: platelet-derived angiogenesis factor
PDGF: platelet-derived grow factor
PEDF: pigment-epithelium-derived factor
Ph: photoreceptors
PlGF: placental growth factor
PLT: Platelets
PRP: platelet rich plasma
Pts: points
RPE: retinal pigment epithelial
RTA: retinal thickness average
SD-OCT: spectral domain-optical coherence tomography
SD: standard deviation
SVF: stromal-vascular fraction
T0: time before of implantation of cell autograft cells
T180: time 6 months post implantation of cell autograft
TGF-β: transforming growth factor-β
TSP: thrombospondin
VEGF: vascular endothelial growth factor
VP: Visual Performance.
